# RNA ligase ribozymes with a small catalytic core

**DOI:** 10.1038/s41598-023-35584-9

**Published:** 2023-05-26

**Authors:** Yoko Nomura, Yohei Yokobayashi

**Affiliations:** Nucleic Acid Chemistry and Engineering Unit, Okinawa Institute of Science and Technology Graduate University, Onna, Okinawa 904-0495 Japan

**Keywords:** RNA, RNA, Origin of life, Nucleic acids, Ribozymes

## Abstract

Catalytic RNAs, or ribozymes, catalyze diverse chemical reactions that could have sustained primordial life in the hypothetical RNA world. Many natural ribozymes and laboratory evolved ribozymes exhibit efficient catalysis mediated by elaborate catalytic cores within complex tertiary structures. However, such complex RNA structures and sequences are unlikely to have emerged by chance during the earliest phase of chemical evolution. Here, we explored simple and small ribozyme motifs capable of ligating two RNA fragments in a template-directed fashion (ligase ribozymes). One-round selection of small ligase ribozymes followed by deep sequencing revealed a ligase ribozyme motif comprising a three-nucleotide loop opposite to the ligation junction. The observed ligation was magnesium(II) dependent and appears to form a 2′–5′ phosphodiester linkage. The fact that such a small RNA motif can function as a catalyst supports a scenario in which RNA or other primordial nucleic acids played a central role in chemical evolution of life.

## Introduction

The RNA world hypothesis proposes that at an early phase of prebiotic evolution, RNA played the role of both a carrier of genetic information and catalysts of various reactions that are needed to sustain primordial life^1–3^. Extant RNA catalysts, or ribozymes, such as group I introns^4,5^, self-cleaving ribozymes^6–9^, and the functional RNA core of the ribosome machinery^10^, are viewed as evidence that RNAs are capable of catalyzing important biological reactions. Furthermore, in vitro selection experiments of ribozymes from random RNA sequences have yielded artificial ribozymes that can catalyze diverse chemical reactions with impressive efficiency^11,12^. However, most of these natural and synthetic ribozymes contain a complex catalytic core within a large structure. Long RNA sequences necessary to encode such ribozymes are not likely to have emerged by chance during the early phase of chemical evolution. It is likely that simpler and smaller, perhaps less efficient, ribozymes played crucial roles in primordial life before the evolution of complex and more efficient ribozymes.

In this work, we explored such simple ribozymes that can catalyze template-directed ligation of RNA fragments. Specifically, we selected ribozymes that can catalyze the formation of a phosphodiester linkage between a free 3′ terminus of an RNA fragment and a 5′-triphosphorylated RNA fragment from a small RNA library with 7 or 8 randomized nucleotides. We discovered a small catalytic core comprising a three-nucleotide motif located opposite to the ligation junction. The ligation yielded a 2′–5′ phosphodiester linkage. Our results suggest that the potential roles of simple RNA motifs with catalytic activity in the origin of life deserve further investigations.

## Results and discussion

### One-round selection of ligase ribozymes

**Figure 1:**
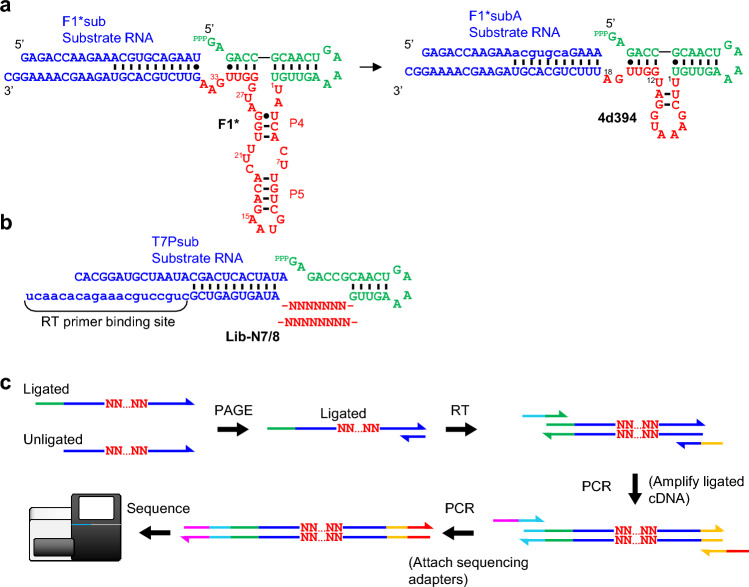
Ribozyme library design and analysis. (**a**) Secondary structures of F1* and 4d394 ligase ribozymes with cognate substrates. Catalytic cores are shown in red. (**b**) Ribozyme library Lib-N7/8. Nucleotides shown in red are randomized. (**c**) Outline of the sequencing library construction. The ligated and unligated RNA strands are first reverse transcribed (RT) to cDNAs and then amplified by PCR. Another PCR step adds adapter sequences for analysis by MiSeq.

In our recent work^13^, we systematically minimized an RNA ligase F1* originally discovered by the Joyce group^14^ through multiple rounds of ribozyme sequence design and high-throughput assay. F1* contained a 35-nt catalytic core which we minimized to 18-nt (4d394, Fig. 1a) through this process. Both F1* and 4d394 catalyzed formation of a 3′–5′ phosphodiester linkage between the substrate fragment and the ribozyme’s triphosphorylated 5′-terminus in a template-directed fashion. Minimization of the catalytic core, however, was accompanied by ~ 860-fold reduction in catalytic efficiency. We decided to search for even smaller catalytic motifs by selecting for ligation activity of a partially randomized ribozyme library depicted in Fig. 1b. Ribozyme library Lib-N7/8 contained a triphosphorylated 5′-terminus connected to a 5-bp stem-loop which is followed by a randomized (N7 or N8) catalytic core. The putative catalytic core is followed by a substrate binding domain and a primer binding site for reverse transcription (Fig. 1b).

Lib-N7/8 contains 81,920 (= 4^7^ + 4^8^) distinct sequences. The library was allowed to react for 4 h at 42 °C in EPPS pH 8.5 buffer supplemented with 25 mM MgCl_2_ with the substrate fragment T7Psub. T7Psub was designed to hybridize with the substrate-binding region of the ribozyme library to place its 3′-terminus in proximity to the 5′ end of the ribozyme. The ligated products were isolated by polyacrylamide gel electrophoresis (PAGE).

The purified ligation products were converted to cDNAs using a reverse transcriptase. The ligated products yield longer cDNAs containing the sequence corresponding to T7Psub on the 3′ end. PCR amplification of the cDNAs using appropriate primers selectively amplifies the ligated sequences. Another PCR step further adds adapter sequences necessary for deep sequencing using Illumina MiSeq (Fig. 1c). Due to the small number of variants in the library, single-round selection was sufficient to enrich and identify catalytically active subsets after deep sequencing. The ten most abundant sequences are listed in Table 1, and the complete sequencing results are provided as Supplementary Data 1. The enriched sequences display some clear trends, for example, all sequences end with “GAU”, with 8 out of 10 sequences ending with “GGAU”.

**Table 1:** The most enriched sequences in Lib-N7/8.

ID	Sequence	Abundance (%)
N8-1	UGGUGGAU	0.642
N8-2	UGUUGGAU	0.377
N8-3	GGGUGGAU	0.354
N8-4	AUGUGGAU	0.342
N8-5	AGGUGGAU	0.340
N8-6	UGGGGGAU	0.268
N8-7	UGGAGGAU	0.262
N7-1	UGAGGAU	0.223
N8-8	UGGGUGAU	0.215
N7-2	GUAUGAU	0.210

**Figure 2:**
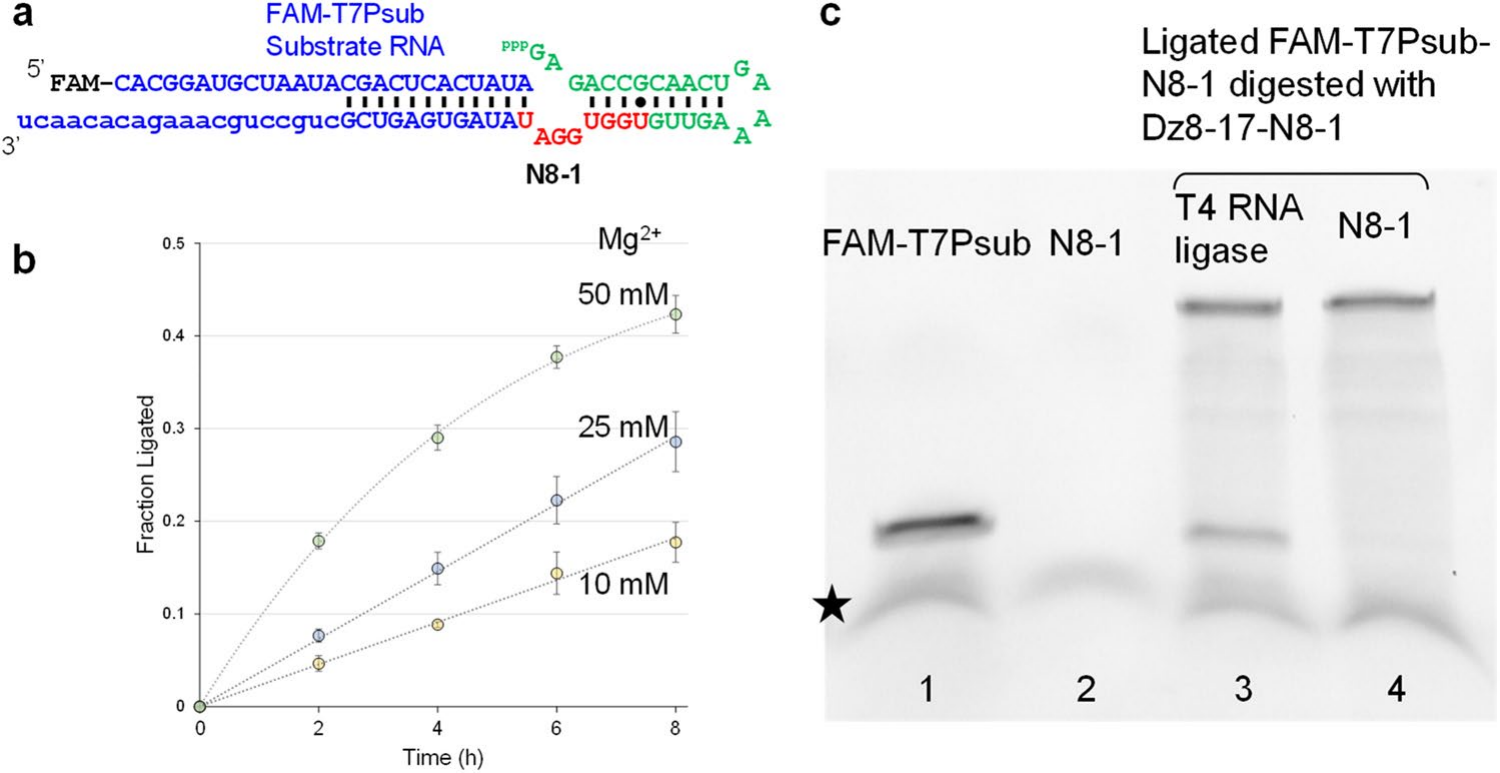
Characterization of N8-1 catalysis. (**a**) Predicted secondary structure of N8-1 in the presence of T7Psub by NUPACK^15^. (**b**) Ligation kinetics of N8-1 with T7Psub in the presence of 10, 25, and 50 mM MgCl_2_. First-order rate constants (*k*_obs_) were estimated by calculating the initial rates of the reactions by fitting the data to linear (10 and 25 mM) or third order polynomial functions (50 mM). The error bars indicate the range of two measurements performed independently. (**c**) Digestion of the N8-1 ligation product by Dz8-17-N8-1 for analysis of the ligation regioselectivity. Lane 1: FAM-T7Psub as a size marker. Lane 2: N8-1 RNA (unlabeled, not visible). Lane 3: FAM-T7Psub-N8-1 ligation product produced by T4 RNA ligase, digested by Dz8-17-N8-1. Lane 4: FAM-T7Psub-N8-1 ligation product catalyzed by N8-1, digested by Dz8-17-N8-1. The lower bands marked by a star are from the loading dye used.

Secondary structure prediction of the most abundant variant (N8-1) in the presence of T7Psub using NUPACK^15^ results in an extended stem-loop, single-stranded GGA, followed by a Watson–Crick interaction with the 3′-end of T7Psub (Fig. 2a). Sequence similarity of the other highly enriched sequences suggests that they also fold into similar structures. The structure suggests that the single-stranded GGA motif between the two substrate binding arms functions as a catalytic core to ligate the 3′ end of the substrate T7Psub with the 5′-triphosphorylated GAG extension upstream of the stem-loop.

### Characterization of the GGA catalytic motif

We confirmed the ribozyme activity of N8-1 by conventional polyacrylamide gel electrophoresis (PAGE). Fluorescently-labeled T7Psub (FAM-T7Psub) was reacted with an excess unlabeled N8-1 to approximate a first-order reaction. The reaction products were sampled at different time points and analyzed by PAGE. Intensities of bands corresponding to the unreacted FAM-T7Psub and the ligated FAM-T7Psub were measured to calculate the fraction of FAM-T7Psub that was ligated (FL: fraction ligated). With 10 mM MgCl_2_, ~ 18% of FAM-T7Psub reacted after 8 h in a linear fashion (*k*_obs_ = 2.3 × 10^−2^ h^−1^) (Fig. 2b). The reaction velocity increased roughly linearly as MgCl_2_ concentration was increased to 25 mM (*k*_obs_ = 3.6 × 10^−2^ h^−1^) and 50 mM (*k*_obs_ = 0.10 h^−1^) (Fig. 2b). No reaction was observed in the absence of MgCl_2_ during the experimental period (data not shown).

Previously identified RNA ligase ribozymes have yielded either 2′–5′ or 3′–5′ phosphodiester linkages. We probed the regioselectivity of N8-1-catalyzed ligation by digesting the ligation product using a deoxyribozyme (DNAzyme) 8–17. DNAzyme 8–17 (Dz8-17-N8.1, Table 3) digests 3′–5′ but not 2′–5′ phosphodiester linkage^16,17^. As a positive control, we synthesized a native 3′–5′ ligation product of FAM-T7Psub and N8-1 using T4 RNA ligase. Treatment of the N8-1-catalyzed ligation product with Dz8-17-N8-1 yielded no detectable cleavage product while the T4 RNA ligase-catalyzed ligation product was clearly targeted by the DNAzyme (Fig. 2c and Fig. S1). Consequently, it is likely that N8-1 yields noncanonical 2′–5′ phosphodiester linkage. FAM-T7Psub analogs with 2′-deoxyadenosine or 3′-deoxyadenosine at the 3′ end did not react with N8-1 under the same conditions (data not shown). Therefore, it appears that the 3′-OH plays some role in accelerating the reaction.

Previous in vitro selection experiments have yielded ligase ribozymes with a 2′–5′ ligation linkage^18–20^. One of the earliest ribozyme selection experiments by Ekland and coworkers yielded 7 families of ligase ribozymes of which 6 catalyzed 2′–5′ ligation^18^. Pitt and Ferré-D’Amaré compared the structures of ligase ribozymes that yield 2′–5′ and 3′–5′ linkages and observed that the conformations of the nucleotides near the ligation junction determines the regioselectivity^21^. Therefore, it is likely that the N8-1 motif places the 2′-OH group of the substrate in a favorable orientation to attack the α phosphorus atom in the 5′-triphosphate group of the ribozyme.

### Reselection of the catalytic core mutants

**Figure 3:**
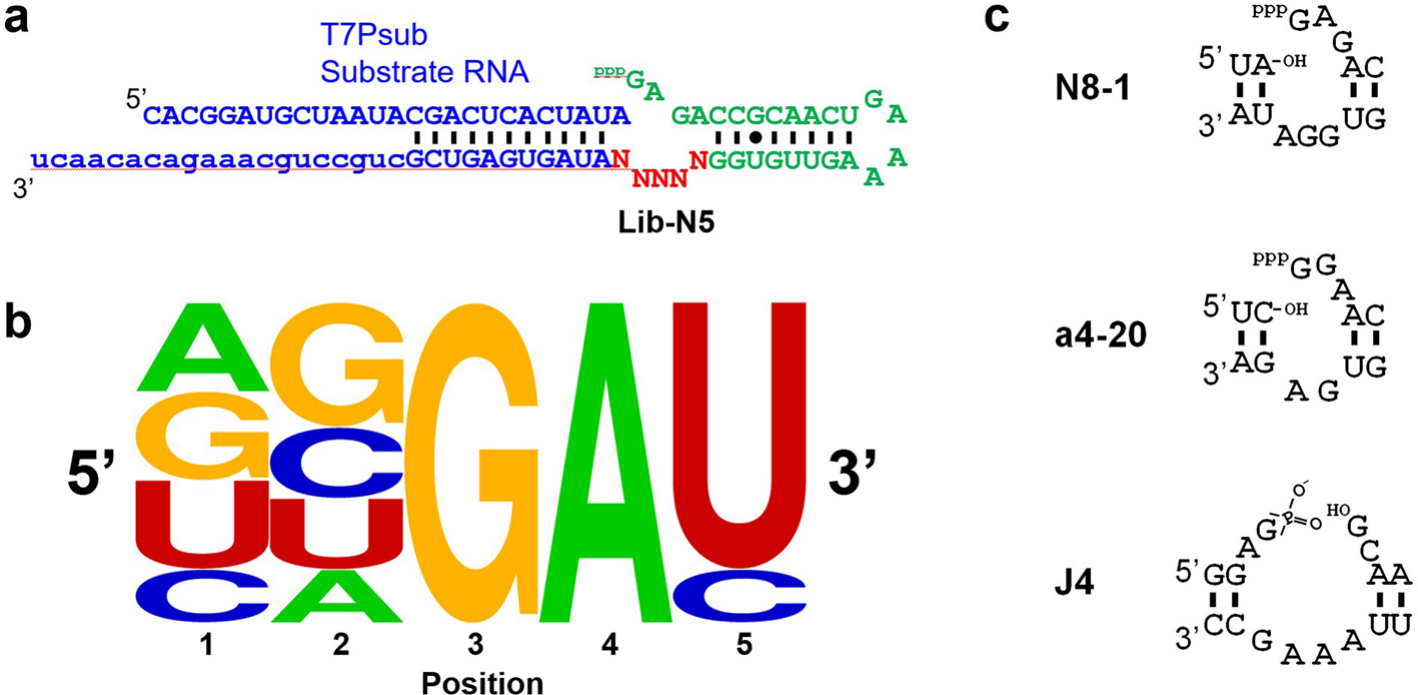
Lib-N5 selection. (**a**) Lib-N5 design. (**b**) Consensus sequence (frequency plot) of the 18 sequences that yielded enrichment of 10 or greater. The logo was generated by WebLogo^22^. (**c**) Ligation junctions of N8-1, a4-20, and J4 ribozymes.

To gain more quantitative insights into the catalytic core of N8-1, a new library Lib-N5 was prepared in which the five nucleotides containing the GGA motif and the flanking nucleotides were randomized (Fig. 3a). One-round selection and sequencing was performed as described above. Moreover, we sequenced the unselected library to quantify the abundance of each variant before the ligation reaction. Enrichment of each of the 1024 variants in Lib-N5 was calculated by dividing the abundance of the variant after selection by that of before selection (Table 2, Supplementary Data 1). As expected, N8-1 (UGGAU) was enriched the most upon selection by 233-fold. Analysis of the 18 sequences that showed enrichment of tenfold or higher suggests NNGA(U/C) as the consensus motif (Fig. 3b). GA at positions 3 and 4 are conserved, and 15 out of the 18 sequences contain U at position 5 with the remaining three sequences containing C. This result further shows that the requirement for catalytic activity is not highly restrictive.

**Table 2:** Enrichment of Lib-N5 variants.

Sequence	Enrichment
UGGAU	233.2
**G**GGAU	166.7
**A**GGAU	95.7
**GU**GAU	89.4
**C**GGAU	57.6
**AU**GAU	34.6
**G**GGA**C**	30.7
U**U**GAU	29.9
UGGA**C**	24.7
**CU**GAU	22.0
**GC**GAU	21.9
**AA**GAU	16.6
**AC**GAU	16.5
**CC**GAU	15.8
U**A**GAU	15.6
**A**GGA**C**	14.4
U**C**GAU	13.3
**GA**GAU	11.7

### Sequence requirements at the 5′ end of N8-1

**Figure 4:**
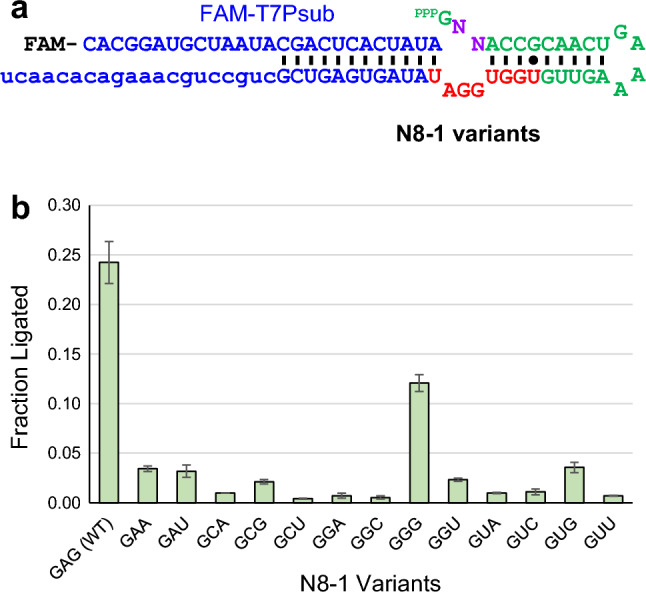
Sequence requirement of N8-1 at the 5′ end. (**a**) Nucleotides mutated (N) in N8-1. (**b**) Ligation yields of N8-1 mutants. The first 3 nucleotides of the mutants are listed. The yields were measured after 4 h reaction at 42 °C in the presence of 25 mM MgCl_2_. The error bars represent the range of two independent measurements.

The first 3 nucleotides GAG of N8-1 do not appear to engage in Watson–Crick base pairing. We varied the second and third nucleotides of N8-1 (GNN) to assess the sequence requirements in the vicinity of the ligation junction (Fig. 4a). The first guanosine was not varied due to the requirement of T7 RNA polymerase used to generate the ribozyme variants. Variants GAC and GCC were also not examined because in vitro transcription reactions yielded unexpected product sizes (data not shown). Out of the 13 variants studied, GGG yielded 12% product after 4 h reaction in 25 mM MgCl_2_ at 42 °C while the N8-1 yielded 24% (Fig. 4b). The other single mutants in the second nucleotide (GCG and GUG) yielded lower (2.1% and 3.6%) but detectable products. Variants GAA, GAU, and GGU also yielded detectable ligation products > 2% (Fig. 4b). Therefore, it appears that these nucleotides play some role in catalysis.

### Small but slow ribozymes

Natural ribozymes such as group I intron have highly evolved and complex structures with high catalytic efficiencies^23^. Similarly, laboratory evolution experiments of artificial ribozymes have mostly focused on optimizing catalytic activity, thus generally resulted in rather complex sequences and structures. Less attention has been paid to smaller and less efficient ribozyme sequences, as such sequences are quickly lost in laboratory evolution experiments. A notable exception is a 5-nucleotide *trans*-aminoacylating ribozyme discovered by the Yarus group. A remarkably small catalytic core comprising 3 nucleotides was shown to catalyze acylation of the 2′-hydroxyl group of a tetranucleotide substrate^24^.

Occasionally, researchers observe residual activity of a small sub-motif of a complex nucleic acid enzyme discovered through in vitro selection. The ligation junction alone of a class II ligase by Ekland et al., (a4-20) was found to catalyze 2′-5′ ligation (Fig. 3c)^18^. While this motif looks intriguingly similar to N8-1, the observed ligation rate was markedly lower (*k*_obs_ = 5.4 × 10^−5^ h^−1^) albeit at different conditions (60 mM MgCl_2_, pH 7.4, 22 °C)^18^. More recently, Mutschler et al., discovered a tetranucleotide loop motif 5′ GAAA 3′ that catalyzes ligation between a fragment containing a 2′,3′ cyclic phosphate terminus with another fragment with a 5′-hydroxyl terminus in a template directed fashion yielding a 2′–5′ ligation linkage^25^. Interestingly, the catalytic motif (J4) and the ligation junction appear somewhat similar to that of N8-1 (Fig. 3c). Apart from the different activation chemistry, the J4 motif showed much lower activity compared to N8-1, for example, yielding ~ 15% ligation product after 5 days under an optimal condition. While selection has played a critical role in the discoveries of these small catalytic motifs, additional deliberate efforts are essential for identification of slow but minimal catalytic cores.

## Conclusion

We identified a new motif comprising only three nucleotides that significantly accelerates templated-directed RNA ligation. Combined with few other examples of similarly small catalytic motifs, it is reasonable to expect other simple RNA motifs exist that can catalyze additional reactions. While these small ribozymes exhibit modest catalytic efficiencies compared to the larger, more extensively engineered counterparts, they could have played important roles in primordial living systems before the emergence of complex machinery that can accurately replicate long RNAs.

## Methods

### Oligonucleotides

The oligo DNAs and RNAs were purchased from Sigma-Genosys or Eurofins with cartridge purification. The sequences are provided in Table 3.

**Table 3:** Oligo DNA/RNA sequences.

Name	Sequence (5′ to 3′)
LibT7P-f	CACGGATGCTAATACGACTCACTATAGAGACCGCAACTGAAAAGTTG
LibT7P-N5-rt3-r	AGTTGTGTCTTTGCAGGCAGCGACTCACTATNNNNNCCACAACTTTTCAGTTGCGGTCTC
LibT7P-N7-rt3-r	AGTTGTGTCTTTGCAGGCAGCGACTCACTATNNNNNNNCAACTTTTCAGTTGCGGTCTC
LibT7P-N8-rt3-r	AGTTGTGTCTTTGCAGGCAGCGACTCACTATNNNNNNNNCAACTTTTCAGTTGCGGTCTC
rt3-r	AGTTGTGTCTTTGCAGGCAG
R2-T7P-f	GTGACTGGAGTTCAGACGTGTGCTCTTCCGATCT-CACGGATGCTAATACGACTCAC
R1-bc2-T7P-rt3-r	ACACTCTTTCCCTACACGACGCTCTTCCGATCTGCGAACTCAAGTTGTGTCTTTGCAGGCAG
TruSeq-i7-UDI0003	CAAGCAGAAGACGGCATACGAGATCCAAGTCCGTGACTGGAGTTCAGACGTGTG
TruSeq-i5-UDI0003	AATGATACGGCGACCACCGAGATCTACACCGCAGACGACACTCTTTCCCTACACGACGC
T7P-N8-1-f	GCTAATACGACTCACTATAGAGACCGCAACTGAAAAGTTGTGGT
N8-1-rt3-r	AGTTGTGTCTTTGCAGGCAGCGACTCACTATATCCACCACAACTTTTCAGTTGCGGT
T7Psub (RNA)	CACGGAUGCUAAUACGACUCACUAUA
FAM-T7Psub (RNA)	FAM-CACGGAUGCUAAUACGACUCACUAUA
Dz8-17-N8-1	TTTCAGTTGCGGTCTTCCGAGCCGGACGAATAGTGAGTCGTAT
R2-T7P-f	GTGACTGGAGTTCAGACGTGTGCTCTTCCGATCTCACGGATGCTAATACGACTCAC
R2-lig-f	GTGACTGGAGTTCAGACGTGTGCTCTTCCGATCTGAGACCGCAACTGAAAAGTTGT
R1-bc1-T7P-rt3-r	ACACTCTTTCCCTACACGACGCTCTTCCGATCTTATCCTCTAGTTGTGTCTTTGCAGGCAG
R1-bc2-T7P-rt3-r	ACACTCTTTCCCTACACGACGCTCTTCCGATCTGCGAACTCAAGTTGTGTCTTTGCAGGCAG
R1-bc3-T7P-rt3-r	ACACTCTTTCCCTACACGACGCTCTTCCGATCTAGAGTAGACAAGTTGTGTCTTTGCAGGCAG
R1-bc4-T7P-rt3-r	ACACTCTTTCCCTACACGACGCTCTTCCGATCTCTCTGGAGTAGTTGTGTCTTTGCAGGCAG

### One-round selection of Lib-N7/8

Libraries with 7 degenerate bases (N7) and 8 degenerate bases (N8) were prepared separately, and they were mixed in 1:4 ratio so that each variant was represented equally in the final library (Lib-N7/8). To prepare the DNA templates for in vitro transcription, 2 μM each of the forward (LibT7P-f) and the reverse (LibT7P-N7-rt3-r or Libt7P-N8-rt3-r) oligos were annealed and extended by OneTaq 2X Master Mix with Standard Buffer (New England Biolabs) in 200 μL volume. The reaction solutions were first denatured at 94 °C for 5 min followed by two cycles of 94 °C for 30 s, 56 °C for 30 s, and 68 °C for 8 s. Afterwards, the solutions were kept at 68 °C for 3 min to ensure complete extension. The DNA templates were purified by DNA Clean & Concentrator-5 kit (Zymo Research).

The library DNA templates were transcribed into RNA libraries using ScriptMax Thermo T7 RNA Polymerase Kit (Toyobo) according to the manufacturer’s instructions. Specifically, the reactions were performed in 10 μL volume using 200 ng of the DNA template. In vitro transcription reactions were performed at 50 °C for 1 h. Subsequently, 10 μL of DNase solution (2 μL Recombinant DNase I (New England Biolabs), 2 μL 10 × DNase I buffer, 6 μL H_2_O) was added and kept at 37 °C for 10 min to digest the DNA template. The transcribed RNA libraries were purified using RNA Clean & Concentrator-5 kit (Zymo Research). The RNA libraries were stored at -80 °C until use.

Eight microliters of the Lib-N7/8 (1.5 μM) RNA was mixed with 4 μL of the substrate T7Psub (24 μM). The mixture was incubated at 70 °C for 3 min and then placed on ice. Four microliters of the 4 × reaction buffer (200 mM EPPS pH 8.5, 100 mM MgCl_2_, 10% RNase Inhibitor, Murine (New England Biolabs) was added to the RNA solution to initiate the ligation reaction for 4 h at 42 °C. The reaction was stopped by addition of 36 μL stop solution (1:2.6 mix of 0.5 M EDTA and 2X RNA Loading Dye from New England Biolabs) and was stored at − 80 °C until gel purification.

The ligation reaction mixture was separated by 8% denaturing PAGE. The ligated products were excised from the gel using a size marker as a guide. The gel fragment was frozen at − 80 °C and crushed. Elution buffer (30 mM Tris–HCl pH 7.5, 30 mM NaCl) was added to the crushed gel to extract the RNA at 4 °C while shaking at 600 rpm for 4 h (ATTO WSC-2630, Power BLOCK Shaker). The extracted RNA was isolated from the gel by a spin column (Vivaclear MINI 0.8 μm, Sartorius), and the RNA was ethanol precipitated using Quick Precip Plus Solution (EdgeBio). The RNA pellet was washed with 70% ethanol and suspended in 10 μL of nuclease free water.

The purified RNA was reverse-transcribed using Maxima H Minus Reverse Transcriptase (Thermo Fischer Scientific) according to the manufacturer’s protocol. Briefly, a mixture of RNA (2.5 μL), 10 μM primer rt3-r (1 μL), 10 mM dNTP mix (0.5 μL), and 3.25 μL H_2_O (7.25 μL total) was incubated at 65 °C for 5 min followed by incubation on ice. Then, a mixture of 2 μL 5 × buffer, 0.25 μL RNase inhibitor, and 0.5 μL reverse transcriptase were added (10 μL total). The reaction was performed at 65 °C for 30 min followed by heat inactivation at 85 °C for 5 min. The resulting cDNA (0.5 μL) was PCR amplified using OneTaq 2X Master Mix with Standard Buffer (New England Biolabs) in a total volume of 20 μL with 0.2 μM primers (R2-T7P-f and R1-bc2-T7P-rt3-r). The reaction solutions were first denatured at 94 °C for 1 min followed by 12 cycles of 94 °C for 30 s, 52 °C for 30 s, and 68 °C for 8 s, followed by a 3 min incubation at 68 °C. The PCR product was diluted 25-fold with water. This diluted product (15 μL) was further amplified using Q5 High-Fidelity 2X Master Mix in a 100 μL reaction with 0.5 μM primers (TruSeq-i7-UDI0003 and TruSeq-i5-UDI0003). The reaction solutions were first denatured at 98 °C for 30 s followed by 20 cycles of 98 °C for 10 s, 69 °C for 30 s, and 72 °C for 10 s, followed by a 2 min incubation at 72 °C. The PCR product was purified using 3% agarose gel and was submitted for sequencing analysis using MiSeq (Illumina).

The sequencing library was analyzed using MiSeq Reagent Kit v3 (Illumina). Single reads for 130 bases were acquired. Raw sequencing data (fastq file) were analyzed by a custom Python script. The reads that contained the expected constant sequences were used to extract the randomized 7 or 8 bases. Only those sequences that yielded quality score of 20 or greater in all randomized positions were retained and counted for analysis. In total, 100,392 reads were obtained.

### Kinetic analysis of N8-1

DNA template for in vitro transcription was prepared as described above using oligo DNAs T7p-N8-1-f and N8-1-rt3-r. The DNA template was used to generate N8-1 ligase RNA by in vitro transcription as described above. An RNA mixture containing 12 μL of 8.2 μM N8-1 ligase RNA and 6 μL of 2.4 μM FAM-T7Psub was incubated at 70 °C for 3 min and then placed on ice. Separately, 6 μL of 4 × reaction buffers (EPPS 200 mM pH 8.5, 40, 100, or 200 mM MgCl_2_, 5% RNase inhibitor) were prepared. The RNA mixture and the 4 × reaction buffers were separately incubated at 42 °C for 3 min before mixing to initiate the ligation reaction (final concentrations: N8-1 ligase 4.1 μM, FAM-T7Psub 0.6 μM, 10, 25, or 50 mM MgCl_2_, 50 mM EPPS, 1.25% RNase inhibitor). The reaction was carried out at 42 °C. At appropriate time points (0, 2, 4, 6, and 8 h), 4 μL from the reaction solution was collected and mixed with 9 μL stop solution on ice. The samples were stored at -80 °C until PAGE analysis.

The samples were loaded onto 8% polyacrylamide gels after heat treatment (95 °C for 3 min). The ligated and unligated susbtrates were imaged and quantified using a Typhoon FLA9500 imager (GE Healthcare Life Sciences). Band intensities were measured by ImageJ to calculate the percentage of the substrates that were ligated at each time point.

### DNAzyme analysis of the ligation regioselectivity

In vitro transcribed N8-1 RNA was treated with Quick CIP (New England Biolabs) to remove the 5′ triphosphate, and then monophosphorylated by T4 polynucleotide kinase (Takara) according to the respective manufacturer’s instructions. The phopsphorylated N8-1 RNA was ligated to FAM-T7Psub by T4 RNA Ligase 1 (New England Biolabs) following the manufacturer’s protocol, and the ligated product was purified by PAGE as described above. This RNA was used as the 3′–5′ linkage control product. Similarly, ribozyme catalyzed products were purified from the reaction between N8-1 and FAM-T7Psub using PAGE.

The (enzyme or ribozyme) ligated RNA (125 fmol) was mixed with 100 pmol DNAzyme (Dz8-17b-N8.2) in a 4 μL solution containing 100 mM KCl, 50 mM Tris–HCl pH 7.5, and 0.25 mM EDTA. The mixture was heated to 95 °C for 1 min, then cooled to 37 °C (0.1 °C/s), kept at 37 °C for 10 min, and finally cooled to 4 °C. DNAzyme catalyzed RNA digestion was initiated by adding 1.33 μL 5 × buffer (125 mM MgCl_2_, 250 mM EPPS, pH 8.5), 0.665 μL RNase Inhibitor, and 0.665 μL of 20 mM spermidine (6.66 μL final volume), and the mixture was incubated at 37 °C for 1.5 h. TURBO DNase (Thermo Fisher Scientific) and its reaction buffer were added to the final concentration of 0.2 U/μL (13.3 μL final volume) and incubated at 37 °C for 30 min to digest the DNAzyme. Finally, 13.3 μL RNA Loading Dye (2X) was added and analyzed by PAGE as described above.

### One-round selection of Lib-N5

Lib-N5 was prepared as described above for Lib-N7/8 except for using oligo DNAs LibT7P-f and LibT7P-N5 for DNA template production. Lib-N5 RNA (0.75 μM) was ligated to T7Psub (6 μM) in a total volume of 16 μL in the reaction buffer (50 mM EPPS pH 8.5 as 5 × buffer, 25 mM MgCl_2_, 2.5% RNase inhibitor) at 42 °C for 4 h. The reaction was stopped by adding 36 μL of the stop solution on ice. The ligated RNA was recovered and processed for sequencing as described above. Two independent experiments (ligation reaction) were performed.

MiSeq analysis was performed essentially as described above (Lib-N7/8). After reverse transcription using rt3-r as the primer, the first PCR was performed with primers R2-T7P-f and R1-bc#-T7P-rt3-r (after ligation) or R2-lig-f and R1-bc#-T7P-rt3-r (before ligation). “bc#” denotes the barcode sequence that identifies “before” or “after” ligation and the two replicate experiments. The second PCR was performed as described above.

The reads were sorted by the barcode and checked for the complete constant sequences. Sequences with all randomized positions yielding quality score of 30 or greater were retained and counted. The total number of reads for the four groups of experiments (“before” or “after”, replicate 1 or 2) ranged from 195,928 to 696,654.

### Ligation assay of N8-1 variants at the 5′ end

DNA templates for in vitro transcription was prepared as described above using oligo DNAs analogous to T7p-N8-1-f with appropriate mutations at the second and third nucleotides of the transcribed ribozyme and N8-1-rt3-r. The DNA templates were used to generate N8-1 variants by in vitro transcription as described above. Ligation reactions were performed as described above, with the final MgCl_2_ concentration of 25 mM at 42 °C. The samples were quenched at 4 h and analyzed by PAGE as described above.

## Supplementary Information

Supplementary Information 1.

Supplementary Information 2.

## Data Availability

Any data not included in the article or Supplementary Data will be made available upon request to the corresponding author.
