# Supplementary material for: RNA ligase ribozymes with a small catalytic core
Source: Sci Rep. 2023 May 26;13:8584. doi: 10.1038/s41598-023-35584-9 (PMC10219994; doi:10.1038/s41598-023-35584-9)
Supplement: Supplementary file 1 — Supplementary Information 1. [file 41598_2023_35584_MOESM1_ESM.pdf]

Supplementary Material

RNA ligase ribozymes with a small catalytic core

Yoko Nomura and Yohei Yokobayashi*

Nucleic Acid Chemistry and Engineering Unit, Okinawa Institute of Science and Technology Graduate University, Onna, Okinawa, 904-0495, Japan

*Email: yohei.yokobayashi@oist.jp

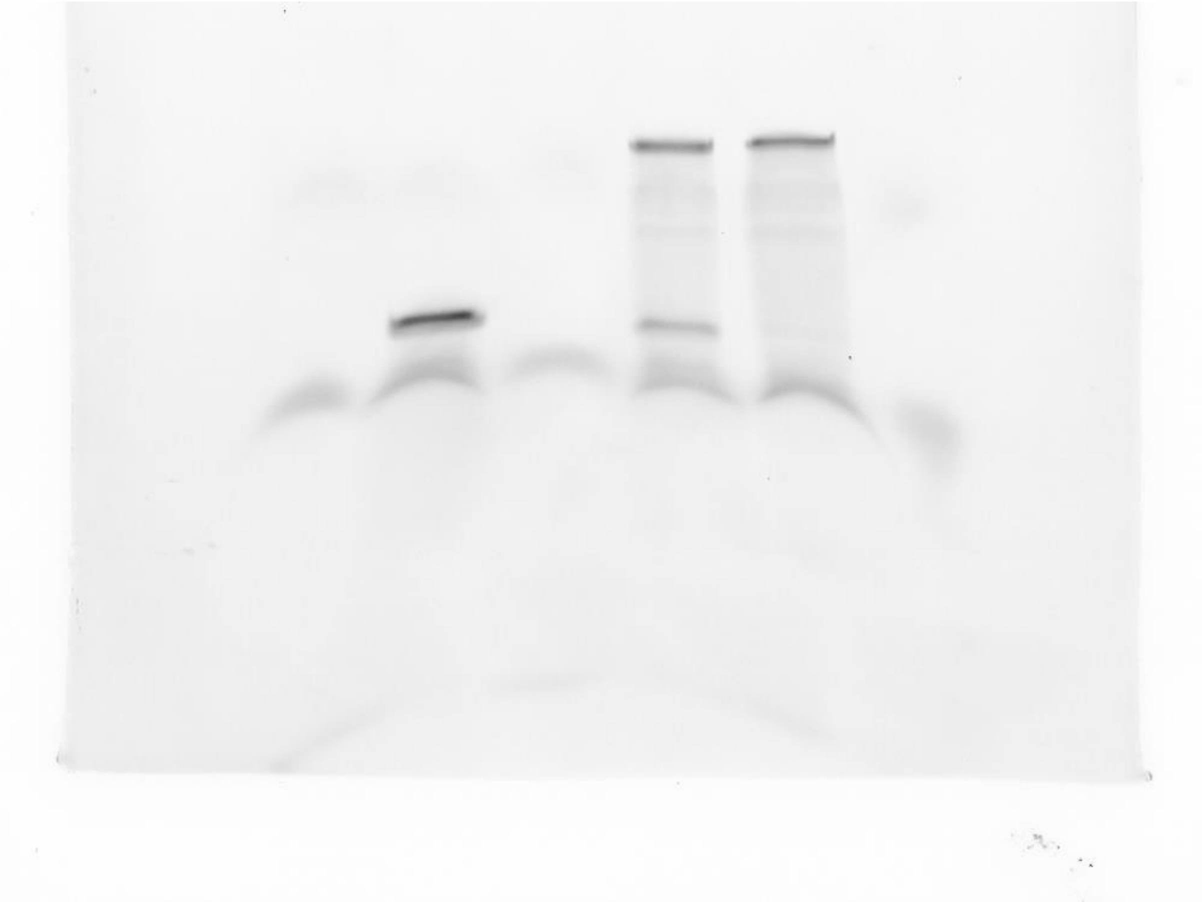

Fig. S1 The original full size gel image of Fig. 2c.
